# 5,8-Dibromo-14,15,17,18-tetra­methyl-2,11-dithia­[3.3]paracyclo­phane

**DOI:** 10.1107/S1600536810026760

**Published:** 2010-07-10

**Authors:** Shuyuan Huang, Qianqian Wang

**Affiliations:** a Key Laboratory of Pesticides and Chemical Biology of the Ministry of Education, College of Chemistry, Central China Normal University, Wuhan 430079, People’s Republic of China

## Abstract

In the title mol­ecule [systematic name: 1^2^,1^5^-dibromo-5^2^,5^3^,5^5^,5^6^-tetramethyl-3,7-dithia-1,5(1,4)-dibenzenacyclooctaphane], C_20_H_22_Br_2_S_2_, the distance between the centroids of the two benzene rings is 3.326 (4) Å, and their mean planes are almost parallel, forming a dihedral angle of 1.05 (7)°. The crystal packing exhibits no inter­molecular contacts shorter than the sum of van der Waals radii.

## Related literature

For the preparation of the title compound, see: Wang *et al.* (2006 ▶). For the crystal structures of related compounds, see: Sun *et al.* (2008 ▶); Clément *et al.* (2009 ▶).
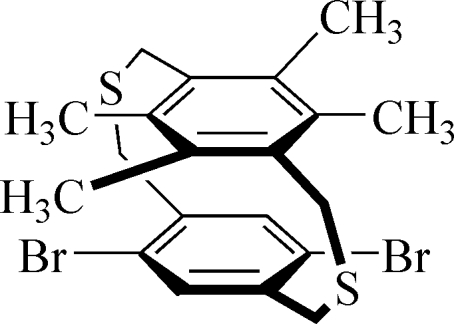


## Experimental

### 

#### Crystal data

C_20_H_22_Br_2_S_2_
*M* *_r_* = 486.32
Monoclinic, 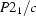

*a* = 15.298 (3) Å
*b* = 12.340 (2) Å
*c* = 10.0160 (18) Å
β = 91.864 (3)°
*V* = 1889.8 (6) Å^3^
*Z* = 4
Mo *K*α radiation
μ = 4.51 mm^−1^
*T* = 298 K
0.23 × 0.20 × 0.20 mm

#### Data collection

Bruker SMART APEX diffractometer
12364 measured reflections
3922 independent reflections
2690 reflections with *I* > 2σ(*I*)
*R*_int_ = 0.075

#### Refinement

*R*[*F* ^2^ > 2σ(*F* ^2^)] = 0.038
*wR*(*F* ^2^) = 0.099
*S* = 0.94
3922 reflections
221 parameters
H-atom parameters constrained
Δρ_max_ = 0.51 e Å^−3^
Δρ_min_ = −0.31 e Å^−3^

### 

Data collection: *SMART* (Bruker, 1997 ▶); cell refinement: *SAINT* (Bruker, 1999 ▶); data reduction: *SAINT*; program(s) used to solve structure: *SHELXS97* (Sheldrick, 2008 ▶); program(s) used to refine structure: *SHELXL97* (Sheldrick, 2008 ▶); molecular graphics: *SHELXTL* (Sheldrick, 2008 ▶); software used to prepare material for publication: *SHELXTL*.

## Supplementary Material

Crystal structure: contains datablocks I, global. DOI: 10.1107/S1600536810026760/cv2738sup1.cif

Structure factors: contains datablocks I. DOI: 10.1107/S1600536810026760/cv2738Isup2.hkl

Additional supplementary materials:  crystallographic information; 3D view; checkCIF report

## Figures and Tables

**Fig. 1.**
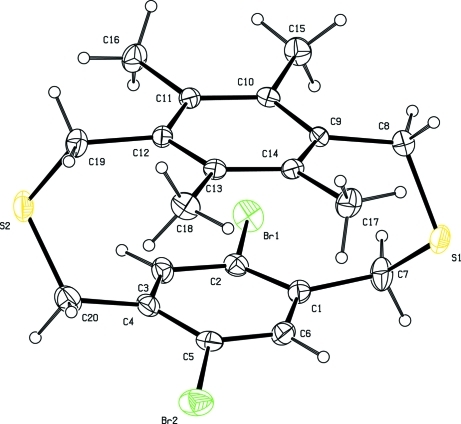
A view of (I), showing the atom-labelling scheme, with displacement ellipsoids drawn at the 50% probability level.

## supplementary crystallographic information

### Comment

As a contribution to a structural studies of paracyclophane compounds (Sun *et al.*, 2008; Clément *et al.*, 2009), we present here the crystal structure of the title compound (I).

In (I) (Fig. 1), the distance between the centroids of two benzene rings is 3.326 (4) Å, and their mean planes are almost parallel forming a dihedral angle of 1.05 (7)°. The crystal packing exhibits no intermolecular contacts shorter than the sum of van der Waals radii

### Experimental

The title compound has been prepared following the known procedure (Wang *et al.*, 2006). A solution with equimolar amounts of 2,5-dibromo-1,4-bis(mercaptomethyl)benzene and 1,4-dibromomethyl-2,3,5,6-tetramethylbenzene in degassed THF(500 mL) was added dropwise under N_2_ over 12 h to a refluxing solution of potassium carbonate(5 equiv) in EtOH(1.2*L*). After an additional 2 h at the reflux temperature, the mixture was cooled and the solvent were removed. The resulting residue was treated with CH_2_Cl_2_(300 mL) and water(300 mL). The organic phase was separated, the aqueous extracted with CH_2_Cl_2_ three times. The combined organic layers was dried over Na_2_SO_4_,then solvent was removed, and the resulting solid was chromatographed on silica gel using CH_2_Cl_2_/petroleum ether(1:1,*v*/*v*) as eluent. The product was further purified by recrystallization from toluene.

### Refinement

All H atoms were initially located in a difference map, but were constrained to an idealized geometry. Constrained bond lengths and isotropic displacement parameters: (C—H =0.93 Å) and *U*_iso_(H) =1.2*U*_eq_(C) for aromatic H atoms, and (C—H =0.97 Å) and *U*_iso_(H) = 1.2*U*_eq_(C) for methylene, and (C—H =0.96 Å) and *U*_iso_(H) = 1.5*U*_eq_(C) for methyl.

### Crystal data

**Table:**

C_20_H_22_Br_2_S_2_	*F*(000) = 976
*M**_r_* = 486.32	*D*_x_ = 1.709 Mg m^−^^3^
Monoclinic, *P*2_1_/*c*	Mo *K*α radiation, λ = 0.71073 Å
Hall symbol: -P 2ybc	Cell parameters from 3874 reflections
*a* = 15.298 (3) Å	θ = 2.6–23.9°
*b* = 12.340 (2) Å	µ = 4.51 mm^−^^1^
*c* = 10.0160 (18) Å	*T* = 298 K
β = 91.864 (3)°	Block, colourless
*V* = 1889.8 (6) Å^3^	0.23 × 0.20 × 0.20 mm
*Z* = 4	

### Data collection

**Table:**

Bruker SMART APEX diffractometer	2690 reflections with *I* > 2σ(*I*)
Radiation source: fine-focus sealed tube	*R*_int_ = 0.075
graphite	θ_max_ = 26.5°, θ_min_ = 2.1°
phi and ω scans	*h* = −12→19
12364 measured reflections	*k* = −15→14
3922 independent reflections	*l* = −12→12

### Refinement

**Table:**

Refinement on *F*^2^	Primary atom site location: structure-invariant direct methods
Least-squares matrix: full	Secondary atom site location: difference Fourier map
*R*[*F*^2^ > 2σ(*F*^2^)] = 0.038	Hydrogen site location: inferred from neighbouring sites
*wR*(*F*^2^) = 0.099	H-atom parameters constrained
*S* = 0.94	*w* = 1/[σ^2^(*F*_o_^2^) + (0.049*P*)^2^] where *P* = (*F*_o_^2^ + 2*F*_c_^2^)/3
3922 reflections	(Δ/σ)_max_ = 0.013
221 parameters	Δρ_max_ = 0.51 e Å^−^^3^
0 restraints	Δρ_min_ = −0.31 e Å^−^^3^

### Special details

**Table:**

Geometry. All e.s.d.'s (except the e.s.d. in the dihedral angle between two l.s. planes) are estimated using the full covariance matrix. The cell e.s.d.'s are taken into account individually in the estimation of e.s.d.'s in distances, angles and torsion angles; correlations between e.s.d.'s in cell parameters are only used when they are defined by crystal symmetry. An approximate (isotropic) treatment of cell e.s.d.'s is used for estimating e.s.d.'s involving l.s. planes.
Refinement. Refinement of *F*^2^ against ALL reflections. The weighted *R*-factor *wR* and goodness of fit *S* are based on *F*^2^, conventional *R*-factors *R* are based on *F*, with *F* set to zero for negative *F*^2^. The threshold expression of *F*^2^ > σ(*F*^2^) is used only for calculating *R*-factors(gt) *etc*. and is not relevant to the choice of reflections for refinement. *R*-factors based on *F*^2^ are statistically about twice as large as those based on *F*, and *R*- factors based on ALL data will be even larger.

### Fractional atomic coordinates and isotropic or equivalent isotropic displacement parameters (Å^2^)

**Table:**

	*x*	*y*	*z*	*U*_iso_*/*U*_eq_	
Br1	0.63291 (3)	−0.24847 (3)	0.69722 (4)	0.06216 (15)	
Br2	0.86653 (3)	0.03415 (3)	1.12714 (4)	0.06978 (17)	
C1	0.7941 (2)	−0.1625 (2)	0.8090 (3)	0.0407 (8)	
C2	0.7035 (2)	−0.1602 (2)	0.8134 (3)	0.0398 (7)	
C3	0.6607 (2)	−0.0921 (3)	0.8980 (3)	0.0445 (8)	
H3	0.5998	−0.0911	0.8961	0.053*	
C4	0.7068 (2)	−0.0249 (2)	0.9864 (3)	0.0436 (8)	
C5	0.7961 (2)	−0.0389 (2)	0.9945 (3)	0.0434 (8)	
C6	0.8387 (2)	−0.1043 (2)	0.9067 (3)	0.0448 (8)	
H6	0.8994	−0.1094	0.9134	0.054*	
C7	0.8438 (3)	−0.2189 (3)	0.7015 (4)	0.0579 (10)	
H7A	0.8025	−0.2425	0.6320	0.069*	
H7B	0.8713	−0.2832	0.7396	0.069*	
C8	0.8677 (2)	−0.0337 (3)	0.5289 (3)	0.0493 (9)	
H8A	0.9104	0.0117	0.4863	0.059*	
H8B	0.8339	−0.0700	0.4585	0.059*	
C9	0.8064 (2)	0.0394 (2)	0.6037 (3)	0.0336 (7)	
C10	0.7159 (2)	0.0343 (2)	0.5758 (3)	0.0351 (7)	
C11	0.65953 (19)	0.0939 (2)	0.6546 (3)	0.0344 (7)	
C12	0.69283 (19)	0.1594 (2)	0.7578 (3)	0.0339 (7)	
C13	0.7842 (2)	0.1727 (2)	0.7759 (3)	0.0340 (7)	
C14	0.84008 (19)	0.1132 (2)	0.6990 (3)	0.0351 (7)	
C15	0.6794 (2)	−0.0317 (3)	0.4589 (3)	0.0533 (9)	
H15A	0.6190	−0.0132	0.4423	0.080*	
H15B	0.6840	−0.1075	0.4796	0.080*	
H15C	0.7119	−0.0162	0.3808	0.080*	
C16	0.5615 (2)	0.0846 (3)	0.6279 (4)	0.0524 (9)	
H16A	0.5425	0.0129	0.6503	0.079*	
H16B	0.5482	0.0984	0.5351	0.079*	
H16C	0.5318	0.1366	0.6815	0.079*	
C17	0.9379 (2)	0.1285 (3)	0.7171 (4)	0.0555 (9)	
H17A	0.9518	0.2041	0.7109	0.083*	
H17B	0.9671	0.0894	0.6487	0.083*	
H17C	0.9569	0.1016	0.8032	0.083*	
C18	0.8205 (3)	0.2505 (3)	0.8816 (4)	0.0532 (9)	
H18A	0.8832	0.2463	0.8850	0.080*	
H18B	0.7986	0.2313	0.9671	0.080*	
H18C	0.8028	0.3231	0.8593	0.080*	
C19	0.6313 (2)	0.2168 (3)	0.8503 (4)	0.0478 (8)	
H19A	0.5883	0.2561	0.7959	0.057*	
H19B	0.6647	0.2697	0.9021	0.057*	
C20	0.6574 (3)	0.0609 (3)	1.0634 (4)	0.0615 (10)	
H20A	0.6990	0.1141	1.0978	0.074*	
H20B	0.6310	0.0265	1.1393	0.074*	
S1	0.92690 (6)	−0.13702 (8)	0.62621 (10)	0.0536 (2)	
S2	0.57294 (6)	0.13011 (8)	0.96634 (10)	0.0572 (3)	

### Atomic displacement parameters (Å^2^)

**Table:**

	*U*^11^	*U*^22^	*U*^33^	*U*^12^	*U*^13^	*U*^23^
Br1	0.0655 (3)	0.0568 (3)	0.0643 (3)	−0.01722 (19)	0.0036 (2)	−0.00873 (18)
Br2	0.0857 (3)	0.0680 (3)	0.0542 (2)	0.0013 (2)	−0.0199 (2)	−0.00657 (19)
C1	0.046 (2)	0.0305 (16)	0.0458 (18)	0.0047 (15)	0.0098 (15)	0.0078 (14)
C2	0.049 (2)	0.0323 (16)	0.0383 (16)	−0.0038 (15)	0.0036 (14)	0.0041 (13)
C3	0.042 (2)	0.046 (2)	0.0456 (18)	−0.0015 (16)	0.0131 (15)	0.0034 (15)
C4	0.057 (2)	0.0401 (18)	0.0345 (16)	0.0056 (16)	0.0110 (15)	0.0066 (14)
C5	0.056 (2)	0.0407 (18)	0.0332 (16)	−0.0002 (16)	−0.0047 (15)	0.0063 (14)
C6	0.0415 (19)	0.0441 (19)	0.0489 (19)	0.0059 (15)	0.0015 (16)	0.0058 (16)
C7	0.059 (2)	0.0427 (19)	0.073 (3)	0.0030 (17)	0.021 (2)	−0.0133 (18)
C8	0.050 (2)	0.055 (2)	0.0434 (19)	0.0080 (17)	0.0094 (16)	0.0000 (15)
C9	0.0344 (18)	0.0372 (16)	0.0298 (14)	0.0046 (13)	0.0075 (12)	0.0063 (12)
C10	0.0409 (19)	0.0344 (16)	0.0301 (14)	−0.0028 (14)	−0.0002 (13)	0.0044 (12)
C11	0.0294 (17)	0.0383 (17)	0.0354 (15)	−0.0014 (13)	0.0021 (13)	0.0062 (13)
C12	0.0345 (17)	0.0303 (15)	0.0371 (15)	0.0023 (13)	0.0063 (13)	0.0041 (12)
C13	0.0367 (18)	0.0319 (15)	0.0333 (15)	−0.0031 (14)	−0.0006 (13)	0.0025 (12)
C14	0.0275 (16)	0.0396 (17)	0.0380 (16)	−0.0027 (13)	0.0004 (13)	0.0107 (13)
C15	0.052 (2)	0.057 (2)	0.050 (2)	−0.0062 (18)	−0.0023 (17)	−0.0121 (17)
C16	0.0309 (19)	0.064 (2)	0.062 (2)	−0.0022 (17)	−0.0018 (16)	−0.0051 (18)
C17	0.0326 (19)	0.065 (2)	0.068 (2)	−0.0055 (17)	−0.0016 (17)	−0.0039 (19)
C18	0.058 (2)	0.052 (2)	0.0492 (19)	−0.0119 (17)	−0.0032 (17)	−0.0060 (17)
C19	0.046 (2)	0.0409 (18)	0.057 (2)	0.0046 (16)	0.0119 (17)	−0.0045 (16)
C20	0.080 (3)	0.061 (2)	0.044 (2)	0.009 (2)	0.0095 (19)	−0.0028 (17)
S1	0.0425 (5)	0.0564 (6)	0.0626 (6)	0.0154 (4)	0.0150 (4)	0.0023 (4)
S2	0.0486 (6)	0.0601 (6)	0.0643 (6)	0.0107 (5)	0.0255 (5)	0.0004 (5)

### Geometric parameters (Å, °)

**Table:**

Br1—C2	1.904 (3)	C11—C16	1.519 (4)
Br2—C5	1.909 (3)	C12—C13	1.413 (4)
C1—C6	1.378 (4)	C12—C19	1.517 (4)
C1—C2	1.388 (4)	C13—C14	1.381 (4)
C1—C7	1.509 (5)	C13—C18	1.521 (4)
C2—C3	1.374 (4)	C14—C17	1.514 (4)
C3—C4	1.389 (5)	C15—H15A	0.9600
C3—H3	0.9300	C15—H15B	0.9600
C4—C5	1.377 (5)	C15—H15C	0.9600
C4—C20	1.524 (5)	C16—H16A	0.9600
C5—C6	1.373 (5)	C16—H16B	0.9600
C6—H6	0.9300	C16—H16C	0.9600
C7—S1	1.808 (4)	C17—H17A	0.9600
C7—H7A	0.9700	C17—H17B	0.9600
C7—H7B	0.9700	C17—H17C	0.9600
C8—C9	1.516 (4)	C18—H18A	0.9600
C8—S1	1.826 (3)	C18—H18B	0.9600
C8—H8A	0.9700	C18—H18C	0.9600
C8—H8B	0.9700	C19—S2	1.833 (4)
C9—C14	1.405 (4)	C19—H19A	0.9700
C9—C10	1.405 (4)	C19—H19B	0.9700
C10—C11	1.397 (4)	C20—S2	1.807 (4)
C10—C15	1.518 (4)	C20—H20A	0.9700
C11—C12	1.396 (4)	C20—H20B	0.9700
			
C6—C1—C2	116.1 (3)	C14—C13—C18	120.3 (3)
C6—C1—C7	119.9 (3)	C12—C13—C18	120.0 (3)
C2—C1—C7	124.0 (3)	C13—C14—C9	120.3 (3)
C3—C2—C1	121.9 (3)	C13—C14—C17	119.7 (3)
C3—C2—Br1	117.0 (3)	C9—C14—C17	120.1 (3)
C1—C2—Br1	121.0 (2)	C10—C15—H15A	109.5
C2—C3—C4	121.0 (3)	C10—C15—H15B	109.5
C2—C3—H3	119.5	H15A—C15—H15B	109.5
C4—C3—H3	119.5	C10—C15—H15C	109.5
C5—C4—C3	116.4 (3)	H15A—C15—H15C	109.5
C5—C4—C20	124.4 (3)	H15B—C15—H15C	109.5
C3—C4—C20	119.2 (3)	C11—C16—H16A	109.5
C6—C5—C4	121.8 (3)	C11—C16—H16B	109.5
C6—C5—Br2	117.0 (3)	H16A—C16—H16B	109.5
C4—C5—Br2	121.2 (3)	C11—C16—H16C	109.5
C5—C6—C1	121.8 (3)	H16A—C16—H16C	109.5
C5—C6—H6	119.1	H16B—C16—H16C	109.5
C1—C6—H6	119.1	C14—C17—H17A	109.5
C1—C7—S1	114.7 (2)	C14—C17—H17B	109.5
C1—C7—H7A	108.6	H17A—C17—H17B	109.5
S1—C7—H7A	108.6	C14—C17—H17C	109.5
C1—C7—H7B	108.6	H17A—C17—H17C	109.5
S1—C7—H7B	108.6	H17B—C17—H17C	109.5
H7A—C7—H7B	107.6	C13—C18—H18A	109.5
C9—C8—S1	117.1 (2)	C13—C18—H18B	109.5
C9—C8—H8A	108.0	H18A—C18—H18B	109.5
S1—C8—H8A	108.0	C13—C18—H18C	109.5
C9—C8—H8B	108.0	H18A—C18—H18C	109.5
S1—C8—H8B	108.0	H18B—C18—H18C	109.5
H8A—C8—H8B	107.3	C12—C19—S2	116.1 (2)
C14—C9—C10	120.1 (3)	C12—C19—H19A	108.3
C14—C9—C8	120.2 (3)	S2—C19—H19A	108.3
C10—C9—C8	119.8 (3)	C12—C19—H19B	108.3
C11—C10—C9	119.1 (3)	S2—C19—H19B	108.3
C11—C10—C15	120.0 (3)	H19A—C19—H19B	107.4
C9—C10—C15	120.9 (3)	C4—C20—S2	114.4 (2)
C12—C11—C10	120.4 (3)	C4—C20—H20A	108.7
C12—C11—C16	120.5 (3)	S2—C20—H20A	108.7
C10—C11—C16	119.0 (3)	C4—C20—H20B	108.7
C11—C12—C13	119.8 (3)	S2—C20—H20B	108.7
C11—C12—C19	120.2 (3)	H20A—C20—H20B	107.6
C13—C12—C19	119.9 (3)	C7—S1—C8	105.64 (18)
C14—C13—C12	119.7 (3)	C20—S2—C19	105.24 (19)
			
C6—C1—C2—C3	8.7 (4)	C9—C10—C11—C16	−177.5 (3)
C7—C1—C2—C3	−167.8 (3)	C15—C10—C11—C16	4.6 (4)
C6—C1—C2—Br1	−173.4 (2)	C10—C11—C12—C13	5.2 (4)
C7—C1—C2—Br1	10.1 (4)	C16—C11—C12—C13	−176.0 (3)
C1—C2—C3—C4	−2.1 (5)	C10—C11—C12—C19	−175.0 (3)
Br1—C2—C3—C4	179.9 (2)	C16—C11—C12—C19	3.8 (4)
C2—C3—C4—C5	−6.9 (5)	C11—C12—C13—C14	−5.7 (4)
C2—C3—C4—C20	171.0 (3)	C19—C12—C13—C14	174.5 (3)
C3—C4—C5—C6	9.1 (5)	C11—C12—C13—C18	175.4 (3)
C20—C4—C5—C6	−168.6 (3)	C19—C12—C13—C18	−4.4 (4)
C3—C4—C5—Br2	−171.9 (2)	C12—C13—C14—C9	−0.4 (4)
C20—C4—C5—Br2	10.3 (4)	C18—C13—C14—C9	178.5 (3)
C4—C5—C6—C1	−2.5 (5)	C12—C13—C14—C17	179.2 (3)
Br2—C5—C6—C1	178.5 (2)	C18—C13—C14—C17	−2.0 (4)
C2—C1—C6—C5	−6.5 (4)	C10—C9—C14—C13	7.0 (4)
C7—C1—C6—C5	170.2 (3)	C8—C9—C14—C13	−174.0 (3)
C6—C1—C7—S1	−46.5 (4)	C10—C9—C14—C17	−172.5 (3)
C2—C1—C7—S1	130.0 (3)	C8—C9—C14—C17	6.4 (4)
S1—C8—C9—C14	66.8 (4)	C11—C12—C19—S2	70.2 (3)
S1—C8—C9—C10	−114.2 (3)	C13—C12—C19—S2	−110.0 (3)
C14—C9—C10—C11	−7.5 (4)	C5—C4—C20—S2	136.9 (3)
C8—C9—C10—C11	173.5 (3)	C3—C4—C20—S2	−40.8 (4)
C14—C9—C10—C15	170.4 (3)	C1—C7—S1—C8	−69.5 (3)
C8—C9—C10—C15	−8.5 (4)	C9—C8—S1—C7	58.3 (3)
C9—C10—C11—C12	1.4 (4)	C4—C20—S2—C19	−71.1 (3)
C15—C10—C11—C12	−176.5 (3)	C12—C19—S2—C20	58.3 (3)
